# Development of a Psychological Intervention to Address Anxiety During Pregnancy in a Low-Income Country

**DOI:** 10.3389/fpsyt.2019.00927

**Published:** 2020-01-10

**Authors:** Najia Atif, Huma Nazir, Shamsa Zafar, Rizwana Chaudhri, Maria Atiq, Luke C. Mullany, Armaan A. Rowther, Abid Malik, Pamela J. Surkan, Atif Rahman

**Affiliations:** ^1^ Human Development Research Foundation, Gujar Khan, Pakistan; ^2^ Department of Gynecology and Obstetrics, Fazaia Medical College, Islamabad, Pakistan; ^3^ Holy Family Hospital, Rawalpindi Medical University, Rawalpindi, Pakistan; ^4^ Department of International Health, Johns Hopkins Bloomberg School of Public Health, Baltimore, MD, United States; ^5^ Department of Psychological Sciences, University of Liverpool, Liverpool, United Kingdom

**Keywords:** prenatal anxiety, psychosocial intervention, low- and middle-income countries, Thinking Healthy Programme, cognitive behavior therapy

## Abstract

**Background:** One in five women suffer from anxiety during pregnancy. Untreated anxiety is a risk factor for postnatal depression and is associated with adverse birth outcomes. Despite the high prevalence of prenatal anxiety in low- and middle-income countries (LMICs), efforts to develop and evaluate context-specific interventions in these settings are lacking. We aimed to develop a culturally appropriate, feasible, and acceptable psychological intervention for perinatal anxiety in the context of a low-income population in Pakistan.

**Methods:** We conducted this research in Rawalpindi District at the Obstetrics Department of the Holy Family Hospital, Rawalpindi Medical University a government facility catering to a mixture of low-income urban, peri-urban, and rural populations. We used a mixture of research methods to: a) investigate the clinical, cultural, and health-service delivery context of perinatal anxiety; b) select an evidence-based approach that suited the population and health-delivery system; c) develop an intervention with extensive reference documentation/manuals; and d) examine issues involved in its implementation. Qualitative data were collected through in-depth interviews and focus group discussions, and analyzed using framework analysis.

**Results:** Informed by the qualitative findings and review of existing evidence-based practices, we developed the “Happy Mother, Healthy Baby” intervention, which was based on principles of cognitive behavior therapy. Its evidence-based elements included: developing an empathetic relationship, challenging thoughts, behavior activation, problem solving, and involving family. These elements were applied using a three-step approach: 1) learning to identify unhealthy or unhelpful thinking and behavior; 2) learning to replace unhealthy or unhelpful thinking and behavior with helpful thinking and behavior; and 3) practicing thinking and acting healthy. Delivered by non-specialist providers, the intervention used culturally appropriate illustrations and examples of healthy activities to set tasks in collaboration with the women to encourage engagement in helpful behaviors. Feedback from the non-specialist providers indicated that the intervention was acceptable, feasible, and perceived to be helpful by the women receiving it.

**Conclusion:** This new psychosocial intervention for perinatal anxiety, based on principles of cognitive behavior therapy and delivered by non-specialists, has the potential to address this important but neglected condition in LMICs.

## Introduction

Pregnancy is marked by intense physiological and psychological changes and increased vulnerability to anxiety (1). A recent systematic review (2) estimated an overall prevalence of 22.9%, with an upward trend across trimesters (18.2%, 19.1%, and 24.6% in first, second, and third trimester, respectively). Anxiety in pregnancy is associated with previous history of anxiety and depression (3–6), prior miscarriage, stillbirth, elective abortion (4, 7), unplanned and unwanted pregnancy (4), concerns about the child’s health (8), fear of childbirth (9, 10), and stressful life events such as unemployment or changes in social support (3, 5, 6). A distinguishing feature of prenatal anxiety is its strong association with fear of pregnancy outcome and childbirth (8), a phenomenon that has also been observed in studies from low- and middle-income countries (LMICs) (11).

Untreated anxiety during pregnancy is one of the strongest risk factors for postnatal depression (12, 13), increasing the likelihood by about threefold (14). It is also associated with adverse birth outcomes including spontaneous abortion, preeclampsia, preterm delivery, low birth weight (15), and decreased birth length (16). Anxiety during pregnancy impairs mother–child bonding in the early postpartum period (17), lowers the odds of exclusive breastfeeding (18, 19), and results in poor child development trajectories (20).

Systematic reviews from high-income countries (HICs) show that psychological interventions in pregnancy are effective in reducing the negative health effects of anxiety on mother and baby (21, 22). Most promising among these include cognitive behavior therapy (CBT) (23–26), behavior activation (27), antenatal education (28, 29), psycho-education (30), and mindfulness therapies (31, 32). These interventions have been tested in both group (23, 27–29, 31, 32) and individual settings (25, 26, 30), using health professionals such as psychologists, obstetricians, midwives, and clinical social workers as well as non-health professionals such as yoga teachers. The number of sessions typically varies between 6 and 12 sessions, and sessions are delivered weekly or coordinated with routine antenatal appointments (21, 22).

Despite the high prevalence of prenatal anxiety in LMICs, efforts to develop and evaluate context-specific approaches in these settings are lacking (2). In response, we aimed to develop a culturally appropriate, feasible, and acceptable psychological intervention for perinatal anxiety, based on the latest evidence, in the context of a low-income population of peri-urban Pakistan.

## Methods

### Settings

We conducted the research in Rawalpindi District, Punjab Province, Pakistan. Rawalpindi is a mixture of urban, peri-urban, and rural populations. The district’s population is typical of a low-income setting in a developing country with high rates of poverty (up to 25% living on less than US$3.5 per day), high fertility rates of 3.8 births per woman, low levels of female education (less than 45% literate), and large households (6.2 persons per household) including extended and joint families. Women are generally economically and socially dependent on members of their families (i.e., the husband, mother-in-law, and their own parents).

Our study was conducted in the Obstetrics Department of the Holy Family Hospital, Rawalpindi, a public hospital with a catchment population of over 7 million drawn from urban as well as peri-urban and rural areas of the district. On any given day, an average of 250 women from low-resource communities receive free antenatal care.

### Research Design

**Figure 1:**
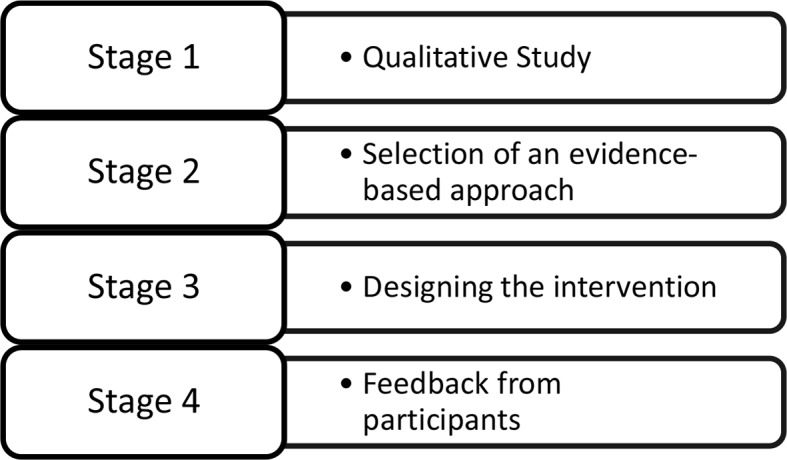
Process of intervention development.

We used a mixture of research methods to: a) investigate the clinical, cultural, and health-service delivery context of perinatal anxiety; b) select an evidence-based approach that suited the population and health-delivery system; c) develop an intervention with extensive reference documentation/manuals; and d) examine issues involved in its implementation (**Figure 1**). Our methods were in accordance with the Medical Research Council (MRC) framework for development and evaluation of complex interventions (33). The framework recommends a development phase to establish the evidence and theoretical basis of an intervention prior to conducting randomized trials to evaluate effectiveness.

#### Stage 1: Conducting the Qualitative Study

We collected data through in-depth interviews with both pregnant women attending the outpatient clinics and health professionals working in the Obstetrics Department. Trained research assistants approached consecutive pregnant women as they attended the department, provided information about the study, and sought consent to participate. Consenting participants were administered the Hospital Anxiety and Depression Scale (HADS) and the Structured Clinical Interview for *DSM-5* (SCID) (34), administered by trained researchers using adapted and translated Urdu versions of the tools. Women scoring positive on the Anxiety scale of the HADS (≥ 8), with no clinical depression or serious medical conditions, and living within 20 km from the hospital were included in the study. The area covered urban, peri-urban, and rural localities and allowed our research participants the convenience to travel to and from hospital for interviews. Purposive sampling was used to recruit health professionals, who included antenatal nurses, midwives, and doctors at Holy Family Hospital. Sampling continued until saturation was achieved.

Separate topic guides (**Table 1**) were developed for the women and health professional participants. They were translated into Urdu, back-translated, and pilot-tested before being administered.

**Table 1:** Areas included in the topic guide.

What do women attribute as the causes of anxiety during the early, mid, and late pregnancy?
How is anxiety expressed both physically and emotionally?
How does anxiety impact day-to-day functioning?
What are pregnant women’s coping strategies used to address anxiety?
What are potential barriers to receiving or delivering a psychological therapy for anxiety, and how could these barriers be overcome?
What factors could facilitate the delivery of a talking therapy for anxiety?

All interviews were recorded, transcribed verbatim, and analyzed using the framework analysis. The framework analysis allows the data to be analyzed systematically in five stages: familiarization, development of the thematic framework or index, indexing, charting, and interpreting the data (35). Researchers and co-authors (NA, PS, SR, and HN) worked in pairs to familiarize themselves with and code the data. Codes were clustered based on their similarities and differences to form themes and subthemes, leading to the development of a thematic framework. The process was overseen by the lead author (NA), and any discrepancies in the findings were resolved through discussion, revisiting the raw data, and referring to the field notes. During this process, the raw data were revisited and indexed in order to revise and finalize the emergent thematic framework. Following this, charts were developed that involved summarizing the indexed data and organizing them under the appropriate theme. All summaries included in the charts were referenced to allow an audit trail of the findings. Finally, we synthesized and interpreted elements of the charts and highlighted key findings.

#### Stage 2: Selecting an Evidence-Based Approach

Following the qualitative study, we adapted the distillation and matching method described by Chorpita and colleagues (2005) (36) to select an evidence-based approach that could be modified for perinatal anxiety. The method recommends drawing a profile of the interventions available for a condition (or related condition), examining their individual elements and strategies, and then matching these to the needs of the new target population based on consideration of their problems, as well as demographic and contextual factors. We reviewed all psychosocial interventions for common mental disorders (largely depression) recommended by the revised version of the World Health Organization’s (WHO’s) Mental Health Gap Action Programme Intervention Guide (mhGAP-IG) (37). The mhGAP-IG was developed through a systematic review of evidence conducted *via* an international consultative and participatory process. It presents integrated management approaches for mental and neurological conditions using protocols for clinical decision-making specifically designed for low- and middle-income settings, where specialists are not widely available. While these WHO psychosocial interventions are indicated for management of depression in the mhGAP-IG, they are often described as transdiagnostic, given the overlap between management of depression, anxiety, and other stress-related conditions. Relevant psychosocial interventions were examined for their theoretical approach, target population, and implementation strategies. An intervention approach best matching the needs of our target population was selected for adaptation.

#### Stage 3: Designing the Intervention

At this stage, an expert panel led by a psychologist (NA), a psychiatrist (AR), and two non-specialist psychology graduates (HN, MA) was assembled to design the intervention. All panel members had experience delivering WHO psychosocial interventions and/or training and supervising others to deliver them to women in the community. Data from the qualitative study and information obtained from the review of WHO mhGAP-IG psychosocial interventions were used to develop a training and supervision manual.

#### Stage 4: Obtaining Feedback From Therapists and Mothers

Once the intervention manuals and procedures were developed, six non-specialist therapists were trained to deliver it by the first author (NA). Training consisted of a 5-day workshop followed by two practice cases of perinatal anxiety undertaken by each trainee under close supervision. Following completion of the therapists’ field training, two focus group discussions were conducted, one with therapists (n = 5) with the aim to explore their experiences of receiving the training and delivering the intervention and one with the pregnant women to explore their experiences of receiving the intervention (n = 5). Both focus groups discussions were recorded and transcribed. Thematic analysis was used to analyze the data. We present descriptive analyses of the feedback.

Ethical approval for the study was obtained from the Rawalpindi Medical University, parent body of Holy Family Teaching Hospital (ref no. IRF/RMC dated 17 December 2016), and the Institutional Review Board (IRB) of the Human Development Research Foundation (ref no. IRB/001/2017 dated 10 March 2017). The study was undertaken from September 2017 to August 2018. All subjects gave written informed consent in accordance with the Declaration of Helsinki.

## Results

### Qualitative Study

In total, 29 in-depth interviews were conducted, 19 with the women with perinatal anxiety and 10 with health-care providers. All interviews were held at the Obstetrics Department. Duration of the interviews was between 45 and 60 min. The mean age of the women was 26 years, and 42% were primigravida. Their years of schooling ranged from no formal education to 11 or more years, with a mean of 4 years. The health professionals interviewed were all experienced, most with over 10 years of working with perinatal women.

Findings were divided into 6 key themes and 18 subthemes. These, along with implications for the intervention, are summarized in **Table 2**. Their description, illustrative quotes, and implications for intervention development are described below.

**Table 2:** Summary of key themes, subthemes, and implications for intervention development.

Themes	Subthemes	Implications for intervention development
Perceived sources of anxiety	Past traumatic experiences Lack of trust in health-care services Not having sufficient support Pressures to produce a male offspring	Be receptive to perceived sources of anxiety; provide information and skills to negotiate health system; involve significant family member in care; gently challenge family attitudes toward male preference
Manifestations and impact of anxiety	Somatic symptoms Emotional symptoms Impact on personal well-being Impact on family	Introduce relaxation techniques such as breathing exercises and meditation;address personal well-being; educate family members about anxiety
Protective factors for anxiety	Adequate antenatal support from professionals Adequate family support Faith and acceptance	Liaison with antenatal services; positively reinforce family support; recognize and support alternate sources of coping such as faith
Desired features of a “talking therapy” for anxiety	Culturally acceptable content and format Appropriate delivery agent Appropriate and convenient venue Involvement of key family members	Use culturally relevant metaphors, idioms, and narratives; female delivery agents; flexible appointments and assistance with transport; engage family members
Potential barriers to intervention delivery	Stigma Lack of empowerment Domestic responsibilities and time constraints	Address stigma; educate and empower; flexible appointment schedule and assistance with transport; engage family members to collaborate in care

#### Sources of Anxiety During Pregnancy

A strong theme emerging from interviews with the mothers was traumatic experiences related to previous pregnancies. These included unplanned or unwanted pregnancy, medical complications related to pregnancy, preterm delivery, and loss of or health problems in the child.

*I am very tense; it’s my 6th pregnancy, and I only had two children alive. My two sons died after 15 and 22 days of birth and one daughter died during pregnancy (miscarriage) (IDI-mother 10).*

A majority of the mothers reported lack of trust in the health services and fear about losing their own or their baby’s life during labor/delivery. Most women, unable to afford private treatment, sought care from the public hospital and often struggled to pay for any extra medical tests or medicines prescribed by their doctors.

*When I think about delivery and recall the comments of other patients regarding hospital services, delivery and operation, it scares me. What if I will lose my baby or if I will die? (IDI-mother 11).*

The relatively poor quality of care available in public sector hospitals was also frequently mentioned by health professionals, who attributed it to lack of funding and overburdened health systems.

Another theme that emerged was about the lack of support from husbands or other family members during pregnancy. Many women described not having anyone to talk to about their concerns. This account was supported by the health professionals responsible for their care during delivery.

*The behaviour of their in-laws and husband is often not good towards them; they don’t talk nicely to them, don’t bring them for checkups, don’t take their problems seriously. Of course, all these things have an impact on patients (IDI-gynecologist).*

Another source of anxiety for the women, especially those who already have daughters, was pressure from their husbands and in-laws to give birth to a male offspring.

*The worry during pregnancy is, mostly thinking about (whether it is going to be) a son or a daughter. The people around you are discussing this issue again and again. That is why women get upset. When the mind is upset all the body gets upset (IDI-mother).*

#### Manifestations and Impact of Anxiety During Pregnancy

A key theme in this area was the range of somatic symptoms experienced by women, including feeling that their “heart was sinking,” palpitations, breathlessness, sweating, dizziness, restlessness, weakness, feeling drained, trembling, numbness, aches, and pains. Women largely attributed these symptoms to a physical cause, which led to concerns about their own and the baby’s well-being.

*When I am anxious, my body starts shivering, Blood pressure gets high. I have a headache, and can’t sleep (IDI-mother 14).*

The main emotional manifestations included feeling worried, fearful, apprehensive, mentally disturbed, tense, angry, withdrawn, uneasy, or tearful.

*Different crazy thoughts come in my mind. I feel so fearful not knowing what will happen to me or my baby (IDI-mother 17).*

With respect to the impact of anxiety, the main themes that emerged were related to the women’s personal well-being, their relationship with people around them, and their relationship with their children. An obstetrician with over 25 years of work experience stated:

*When any woman is psychologically disturbed, her wellbeing gets affected, her diet gets affected, health is also affected. Sometimes her labor also gets effected, as her pain threshold gets lower and the labor can be prolonged, and also the post-delivery recovery takes longer. Most of time she keeps lying in bed, won’t be able to feed her child properly because of lack of support (IDI-gynecologist).*

#### Protective Factors for Anxiety During Pregnancy

Many of the health professionals and the women reported feeling that availability of adequate antenatal care and an empathetic attitude from health professionals could help ease the expectant women’s anxiety and motivate them to attend antenatal checkups.

*If we give individual attention to the patient and give some extra time to them, their anxiety could be treated. Instead of medicines, we need to talk to them and give some counseling to them (IDI-gynecologist 4).*

Another strategy that appeared to protect some women from anxiety was turning to faith, using prayers and religious rituals to deal with their symptoms.

*All I do is to separate myself from everyone and offer my prayers to God. This makes me stay relaxed and calm (IDI-mother 9).*

In terms of protective factors, support from family and friends emerged as a strong theme. This included both direct personal support and support from a distance through social media or mobile phones. Women described that talking to family and friends, in person or through social media, and spending quality time with their children helped them cope with their symptoms of anxiety.

#### Desired Features of a “Talking Therapy” for Anxiety

A majority of the women (18/19) agreed on the potential for a talking therapy to help with their anxiety. They felt that someone who listened to their problems, tried to understand them, and provided suggestions that could make them feel better would be very welcome and that they would find time to engage with such a person. A majority of the participants expressed a need for an intervention which was relevant to their everyday lives and day-to-day problems, helped with their well-being, and instilled hope.

*It will be beneficial, it will increase awareness and give women an opportunity to share their concerns, which could help them to offload (IDI-mother 7).*

A strong theme that emerged in this area was the preference for someone who was empathetic, caring, and courteous, irrespective of whether they were health professionals or not. There was general agreement that such a person should be female and properly trained and supervised.

*It is not important that the person is doctor, midwife or anything; what matters is that the person is a woman, who can listen to other women and who is kind-hearted. I think it is better to train women and then deliver the programme through them (IDI-mother 10).*

There were conflicting views among the women regarding the preferred format of the sessions. Some suggested that a group setting for intervention delivery would be optimal, as it would provide an opportunity to meet and share their problems with other women, while others felt that individual sessions are preferable, as problems could be shared confidentially without feeling embarrassed in front of others.

With regard to session timings, duration, and venue, it was suggested that sessions should be held during the morning, should not be more than an hour, and should be held at the hospital or another health facility. Delivering the talking therapy at the same visit as when the woman attended her routine antenatal appointments was suggested as a way to improve attendance.

*Hospitals are better as women already come here. It will not be possible to receive intervention at homes as there is no privacy there (IDI-mother 16).*

A majority of the women were poor and dependent on their family members for travelling to the hospital. Some suggested that reimbursing travel expenses to attend the therapy sessions could be an attractive incentive; others suggested providing free medicines or supplements.

*If we give mothers information about their health and help them with travel money or supplements they can’t afford, they will be more motivated (IDI-midwife).*

Participants felt that involving the mothers’ significant family members, such as husbands and mothers-in-law, could facilitate their engagement with the intervention. They would need to be involved from the time of recruitment and throughout delivery of the sessions to ensure they supported the mothers in this challenging period.

*Husbands should be aware of his wife’s condition and they have to take care of the needs of their wives. Women can’t do anything until their men understand the problems of pregnancy (IDI-mother 15).*

#### Potential Barriers to Intervention Delivery

##### Stigma of Mental Illness

The lack of awareness about mental illnesses and the stigma attached to them, often preventing disclosure, emerged as a theme that could hinder treatment seeking in some women. Many women felt that they would be labeled as psychiatric patients and mentally weak. They reported fears of being mocked by others or bringing shame to their families. One obstetrician equated the stigma of mental illnesses with the stigma of having serious physical ailments such as cancer and tuberculosis.

*Mental disorders, cancer and TB, if these 3 things have happened to the patient or in her family, they don’t disclose easily. They think it is something which will bring shame to their family (IDI-gynecologist).*

##### Lack of Financial Empowerment

A majority of the participants reported lack of empowerment and financial dependence on their husbands and in-laws. Consequently, they struggled to comply with doctors’ suggestions regarding their treatment and self-care unless their family supported them.

*Sometimes they have lots of financial problems, because of which, like whatever suggestions we give to them, their husbands and in laws don’t comply with them. Sometimes their mothers-in-law say we delivered all our children at home and it was fine so no need to go to antenatal appointments, so they have no choice other than to listen to them (IDI-gynecologist).*

##### Time Constraints

Many women felt overburdened by excessive demands and responsibilities at home, which they felt could prevent them from visiting hospital to attend the sessions. This was validated by the midwives who acknowledged women’s excessive domestic responsibilities as a barrier to attending antenatal appointments.

*God knows how they get time out even for their checkups. It will be difficult for them to come to the hospital to receive intervention. They don’t come even when given an appointment for ultrasound (IDI-nurse).*

### Selection of an Evidence-Based Approach to Address Anxiety

We wanted to select an evidence-based psychosocial approach suitable within the context of LMICs, and hence our target population. This context is characterized by large populations (hence large number of women in need of care), lack of mental health specialists (hence approaches amenable to task-sharing), stigma of mental disorder, lack of literacy, and the key role of family. The psychosocial approaches contained within the WHO mhGAP-IG cater to these needs. We therefore limited ourselves to examining the three psychosocial interventions contained within the WHO mhGAP-IG. These are reviewed and are summarized below.

#### Problem Management Plus

Problem Management Plus is based on problem solving and behavioral techniques and targets adults suffering from symptoms of common mental health problems (e.g., depression, anxiety, stress or grief), as well as self-identified practical problems (e.g., unemployment, interpersonal conflict) (38). One of the core features underpinning PM+ is adherence to a transdiagnostic approach. Transdiagnostic treatments are “those that apply the same underlying principles across mental disorders, without tailoring the protocol to specific diagnoses” (39). The initial session orients participants to the intervention with motivational interviewing techniques to improve engagement, provides information about common reactions to adversity, and teaches participants a basic stress management strategy (slow breathing). The second session addresses a participant-selected problem using problem-solving techniques and introduces behavioral activation, during which an individual is encouraged to gradually reengage with pleasant and task-oriented activities to improve his/her mood and functionality. The third and fourth sessions support participants’ continued application of problem solving, behavioral activation, and stress management and introduce strategies to strengthen social support networks. In the final session, education about retaining treatment gains is provided, and all learned strategies are reviewed. PM+ is intended to be delivered by lay helpers who have completed at least high school but may lack previous mental health training. PM+ has an 8-day training program, followed by a 2- to 3-week period of in-field practice with ongoing, weekly supervision. Supervision is conducted by skilled mental health professionals who have received PM+ training and have experience in its delivery. Additional intervention details are available on the WHO website (38).

#### Group Interpersonal Therapy

Interpersonal psychotherapy (IPT) has demonstrated efficacy for the prevention and treatment of perinatal depression and has also been found to reduce symptoms of anxiety and improve relationship quality, social adjustment, and social support in the perinatal period (40). In the WHO mhGAP group IPT approach, the target population is adults with depression, and lay facilitators help group members to find links between depression and current life problems and to build communication and other interpersonal skills to manage their problems more effectively. The interpersonal nature of the group and the conversations within the group are helpful aspects of this process, because this is where group members learn and where they get ideas about how to address their problems. Once the participant has an idea on how to address a problem, they are encouraged to try it out and then discuss the results in the following session. This may help the person and other group members progressively come up with more helpful ideas to address their problems. The intervention is organized into eight group sessions with 6–10 members per group, and each session lasts 90 min. The intervention can be delivered by supervised facilitators who may not have received previous training in mental health. The training is generally 8–12 days and conducted by experienced mental health professionals. Intervention details are available on the WHO website (41).

#### Thinking Healthy Programme

The Thinking Healthy Programme (THP) was developed specifically within the context of perinatal depression and distress in LMICs and designed to be integrated into maternal and child health programs. It employs specific as well as non-specific elements of CBT, such as building an empathetic relationship, focusing on the here and now, behavior activation, and problem solving. The principles underpinning CBT are delivered in three simplified steps: a) identifying ways of thinking—a mother’s thoughts, beliefs, ideas, attitudes, assumptions, mental imagery, and ways of directing her attention are analyzed with the help of pictures, and unhelpful styles of thinking are identified; b) altering unhelpful styles of thinking—helping the mother make the link between unhealthy thoughts and emotions that can lead to unhelpful behaviors and attempting to replace them with more helpful styles of thinking and acting; and c) practicing healthy behaviors—by achieving the above, helping the mother to practice meeting the challenges and opportunities of raising her baby with a clear and calm mind. The program is fully documented through a comprehensive set of manuals and includes culturally appropriate pictorial illustrations aimed at helping mothers reflect on their thinking processes and at encouraging family support. The sessions are organized into five modules covering the period from the third trimester of pregnancy to 1 year postnatal. Each module focuses on three key areas—the mother’s personal health, the mother–infant relationship, and the psychosocial support of significant others. In total, 16 home-based sessions are offered to mothers. THP is designed to be delivered by community health workers or their equivalent. No prior mental health experience is necessary, but they work under close supervision of specialists with CBT experience. The Thinking Healthy intervention is available on the WHO website (42).

Following an analysis of the elements and strategies employed in these three interventions, and matching these to the needs of the target population, we chose by consensus the THP as the best match for a treatment approach to address anxiety in the perinatal period. The reasons for this were: a) THP is already designed specifically for women in the perinatal period; b) it is based on cognitive and behavioral therapy which has the strongest evidence base for treatment of anxiety; c) the focus on family support, behavioral activation directed toward personal well-being, and problem solving matched the needs of our target population; d) the use of pictures and vignettes to explain thinking and emotions suited the needs of illiterate women; e) the mother-and-child dyad as the focus rather than the mother alone helps engage the entire family and reduce stigma; and f) the THP had been demonstrated to be feasible for delivery by non-specialists as well as acceptable in diverse cultures (43). The expert group examined each element of the THP for its relevance to anxiety and distilled these elements into the new intervention. The group also examined strategies used in the other interventions, especially the transdiagnostic PM+, and included stress management techniques such as breathing exercises, some strategies for relapse prevention, and the progress monitoring tool [Psychological Outcome Profiles (PSYCHLOPS)] in our anxiety intervention. We did not include any elements of interpersonal therapy because the THP already contained elements related to social and family support that were relevant to the context of anxiety problems in our participants.

### Designing the “Happy Mother, Healthy Baby” Intervention for Perinatal Anxiety

We employed a similar format and design as the THP for perinatal depression but adapted it for anxiety. The core strategies of the THP—empathetic relationship building, thought challenging, behavior activation, problem solving, and family involvement—were retained, but the messages were modified in light of our qualitative findings. The cognitive behavioral elements of thought challenging, behavior activation, and problem solving were applied using the same three-step approach of the THP: 1) learning to identify unhealthy or unhelpful thinking and behavior; 2) learning to replace unhealthy or unhelpful thinking and behavior with helpful thinking and behavior; and 3) practicing thinking and acting healthy. The intervention used culturally appropriate illustrations and examples of healthy activities to set tasks in collaboration with the women to encourage engaging in helpful behaviors. We call the intervention “Happy Mother, Healthy Baby (HMHB).”

#### Content of Sessions

HMHB has six core sessions and two to six booster sessions designed to be delivered individually at a health facility or at the client’s home (**Table 3**). The length of the core sessions is 60 min, and the booster session 30 min. The first five core sessions are delivered weekly, starting as early in the pregnancy as possible. These are followed by the booster sessions, the frequency and duration of which could be tailored to individual needs of the woman and integrated into her routine visits. The final core session is delivered late in the third trimester of pregnancy. The first session aims to build rapport with the woman and sets ground rules to ensure her engagement and raise her psychosocial awareness. The second session introduces the three steps of thinking and acting healthy and explains how the woman can apply these steps to manage stress and improve personal well-being. The third session aims at improving the woman’s social support; during this session, attendance of significant family members (such as her husband and/or mother-in-law) is encouraged, the woman maps her social support network, and support for her during pregnancy and after childbirth is negotiated with key family members. The fourth session focuses on the woman’s attachment with her unborn baby. The woman is encouraged to list her anxieties in relation to the labor and delivery process, and each one is discussed. The fifth session involves reviewing the previous sessions to help the woman gauge the level of her anxiety and develop a plan for maintaining any helpful practices learned in previous sessions. The booster sessions are delivered between session 5 and the final core session. They are coordinated with the routine antenatal appointments and aim to reinforce the health messages and encourage use of problem management strategies to deal with any ongoing or new issues closer to the time of delivery. The final core session discusses problems the woman might face in the postnatal period and identifies techniques learned in the previous sessions to help manage them. Families are invited to attend sessions 1, 3, and 6.

**Table 3:** Structure of the Happy Mother, Healthy Baby.

Sessions	Timing
Session 1: Starting the journey to a “Happy Mother, Healthy Baby”	Early in pregnancy (soon after first antenatal visit*)
Session 2: What makes a Happy Mother, Healthy Baby—Your Wellbeing	After 1 week
Session 3: What makes a Happy Mother, Healthy Baby—Your Relationship With People Around You	After 1 week
Session 4: What makes a Happy Mother, Healthy Baby—Your Bond With the Baby	After 1 week
Session 5: What makes a Happy Mother, Healthy Baby—Staying Well	After 1 week
Booster sessions (two to six sessions): What makes a Happy Mother, Healthy Baby—Staying Well	Coordinated with routine antenatal visits
Session 6: What makes a Happy Mother, Healthy Baby—Preparing for the Baby’s Arrival	In the third trimester of pregnancy

*The first antenatal visit must occur at ≤ 22 weeks of pregnancy.

Progress is monitored in each session by reviewing an anxiety chart (consisting of a visual scale of the emotional state represented by five different facial expressions ranging from “very anxious” to “calm”) and administering the PSYCHLOPS (44), a monitoring tool used in PM+ to record the women’s subjective improvement in her progress (**Data Sheet 1**). In cases where the participant is consistently very anxious, progressively worse on the PSYCHLOPS, or displays any ideas or risk of self-harm, discussion with the supervisor is initiated, and the woman may be referred to specialist care.

#### Training and Supervision

The intervention was delivered by non-specialists who had completed at least bachelor’s degree but had no previous mental health training. They received 42–50 h of classroom training delivered by the lead trainer/s, focusing on understanding anxiety and its impact, counseling skills, key principles of CBT, intervention contents and their delivery procedures, and use of monitoring protocols. Following successful completion of the classroom training, the participants underwent field training consisting of two practice cases per trainee to gain firsthand experience delivering the intervention. During the field training, the therapist delivered six core sessions weekly to two women with perinatal anxiety with supervision by their trainers. The sessions were delivered at the Holy Family Hospital.

### Feedback From Participants

Following the practice cases, women (n = 5) and therapists (n = 5) provided feedback to the research team through focus group discussions. All therapists were asked about their experiences of training, supervision, and problems encountered in delivering the intervention. Women were asked about the appropriateness of the intervention, whether they found it useful, and difficulties in following any of the content delivered. The feedback is summarized below along with illustrative quotes.

#### Feedback From Therapists

A majority of the therapists felt that the combination of classroom and field training was adequate and gave them sufficient confidence to deliver the intervention independently. Role play during training was considered effective, but some therapists suggested that the training duration could be extended in order to reduce any apprehensions later, when taking on real cases.

*I think the duration of the training could have been a bit longer. On the final day we had to rush through a few things. It would have been good to have an extra day to practice some skills.*

*I think the classroom training needs to incorporate mock practice sessions. It gave us good command on the contents, but we were not sure how we would implement a whole session in practice.*

The therapists received weekly supervision during their field training, most of which was conducted remotely over Skype to mimic real-life situations in most LMIC settings where trained and experienced supervisors are scarce and normally located in urban centers.

*Delivering this intervention was a new experience for us; we were not sure if we were doing it right or not. Discussing it with the supervisor gave us the clarity and also suggestions if we need to do it differently in our next session.*

While all the therapists found these supervision sessions extremely helpful by allowing them to discuss their cases, validate their newly developed skills, and address any challenges, some felt they missed out on the value of face-to-face supervision.

*We have supervisions over the Skype, therefore we miss out on the physical presence of our supervisor. Although we have been told by our supervisor that we can get in touch with her if anything needs to be discussed urgently, however if she would have been here, we could have discussed matters on a daily basis.*

The therapists felt that their clients engaged with them well and were receptive toward the content of the intervention. The therapists found the pictorial job aids (e.g., anxiety scale and health diary) and structured instructions as well as easy record-keeping tools for each session helpful in delivering the key messages.

*We gave the Health Diary to the mothers to keep a record of how she felt and activities she undertook, and we had Case Files (to assist record-keeping) to make notes of each session. The Case Files were very helpful as we could see our notes and recall our previous session before each appointment. The Reference Manual is really good and essential too, as reading the session’s contents prepares you to deliver the session.*

All the therapists agreed that the illustrations and stories were a very powerful way to raise psychosocial awareness and to help identify unhelpful thoughts and behavior. The women could relate to the characters in the illustrations, and this helped them follow the content of the intervention with relative ease.

*Rashda’s character was very engaging, and the illustrations took them (the participating women) to the next level. It helped them to identify their unhealthy thoughts and helped them to understand how getting rid of them can be beneficial for them.*

#### Feedback From Women

All participating women agreed the intervention was useful and improved their well-being, and they provided many specific examples of which components were most helpful. The main themes included having a greater awareness of their feelings, better management of stress, and a structured way to care for their well-being.

*I feel fresh now. I used to feel scared thinking of the labour room and the delivery, but now I have been able to overcome my fear.*

*I got a lot of benefit from engaging in the healthy activities suggested by her (the therapist), I used to feel sluggish in my previous pregnancies, but now I am feeling active and energised. If I don’t feel like eating, I look at my diet chart in the health file which motivates me to eat.*

The women felt the intervention was acceptable to their family members and led to an increase in the support offered by key family members.

*When my husband attended the session, he gained a lot of information and understood essentials, which otherwise he would never have learned.*

Not all women received the support they needed, especially from husbands, who were either too busy or felt this was not their domain or responsibility.

*I can’t share my issues with my family. They don’t care about me, they don’t help me with the activities or remind me to do them or are willing to accompany me to the hospital.*

The women also complained about overwork; they discussed the burden of household chores and providing supervision and care for young children in extended families. This results in very little time for them, and they often asked for flexibility within their scheduled appointments to allow them to attend the sessions.

The non-specialist therapists were appreciated by all the participants. Participants expressed confidence in their abilities and felt they did a good job.

*She was good in every possible way, she was good at her job, the way she talked and behaved. She encouraged me, helped me get support from my family members, have my routine check-ups, spend quality time with my family and not to worry too much, as it could impact the baby.*

Some participants identified a number of challenges or barriers they faced in receiving the intervention or benefitting fully from it. While there were advantages in integrating the intervention within antenatal care, some drawbacks were highlighted. For example, one woman suggested that the overcrowded and chaotic obstetric department was anxiety provoking, and therefore was not the ideal environment in which to have the session.

*The way things happen in this hospital, having tests and ultrasounds being done is not easy. I was feeling anxious during the session because it is so noisy and chaotic.*

## Discussion

We have described a psychosocial intervention developed specifically for women with perinatal anxiety in low-income countries. The intervention development process adhered to the MRC (UK) framework for development and evaluation of complex interventions (33). It was informed by the evidence base for what works in perinatal anxiety (21, 22), including:

- A well-established theory underpinning the process of expected change
- Detailed formative research to contextualize the intervention to the target population
- Use of evidence-based elements and strategies from existing well-established approaches

Preliminary feedback from participants indicated that the intervention was acceptable, feasible, and perceived to be helpful by the women receiving it.

Perinatal anxiety is a critical public health priority, given the high prevalence, its tendency to predispose women to subsequent postpartum depression, and the negative impacts on child development and health outcomes. Lack of trained mental health specialists to deliver psychosocial interventions is a major barrier to addressing this problem (45). This simple intervention can be delivered by non-specialists under supervision and thus, if shown to be effective in subsequent large scale evaluations, could address the treatment gap for common mental disorders in LMICs (46).

Similar approaches have demonstrated clinical benefit and utility in high-income settings. For example, an evaluation of the UK’s Improving Access to Psychological Therapies program (IAPT) (47) found a substantial reduction in depression and anxiety in people who attended low-intensity interventions delivered by university graduates with a few months of training. A meta-analysis reviewed similar low-intensity interventions and found these approaches to be effective even for individuals with symptoms of severe depression (48).

It should be emphasized, however, that while such so-called “low-intensity” interventions could play an important role in reducing the treatment gap for common mental disorder in LMICs, they are only one component of a collaborative stepped-care model of service delivery. This intervention was designed to address symptoms of anxiety before these become chronic, severe, and debilitating, necessitating more specialized care in the stepped-care pathway. Such interventions can therefore play an important role in prevention, allowing women to learn strategies for stress management and problem solving before the symptoms become ingrained. This model of care fits well with the staging approach to identification and management of mental disorders advocated by the Lancet Commission on Global Mental Health (49). Women who do not respond to this level of care or who go on to develop more serious symptoms should be referred to and able to access more specialized care.

We limited our study population to women living within a 20 km radius of the General Hospital. While our purposive sampling included women from a range of ages, neighborhoods, and socioeconomic gradients, generalization of our findings to other populations should be done with caution. However, our key findings with regards to the sources, manifestations, and impact of anxiety were remarkably similar to existing literature from a range of different settings and contexts (3–11). In matching our intervention approach with available “psychosocial” interventions, we limited ourselves to the WHO mhGAP-IG. We recognize that the intervention packages within the mhGAP-IG are not specifically designed for anxiety, but are described as “transdiagnostic,” given the overlap between management of depression, anxiety, and other stress-related conditions. We are cognizant that other approaches, such as acceptance–commitment and mindfulness therapies, not covered by the mhGAP-IG, might also contain elements that could potentially be useful for our intervention. Neither did we consider anxiety-specific protocols from HICs, given that matching these to the population and health-system context would be problematic. As these approaches become part of the WHO’s recommendations for LMICs in future versions of the mhGAP-IG, our intervention could be revised to include relevant elements from such approaches.

While our preliminary study suggests that the intervention is acceptable and feasible, we do not have evidence of its effectiveness. In keeping with MRC recommendations (33), we are currently conducting a randomized controlled trial of the effectiveness of the intervention in reducing anxiety in the prenatal period as well as preventing depression postnatally in Rawalpindi, Pakistan (see https://clinicaltrials.gov/ct2/show/NCT03880032). If found to be effective, future research could focus on strategies to scale up such interventions in the perinatal period. It would be feasible to integrate this intervention into the well-established WHO THP rather than as a stand-alone intervention, given the similar approach adopted and the overlap between anxiety and depressive symptoms in such settings (50). Such integration would also be important because the same non-specialist therapists are likely to provide such interventions to perinatal women, and it would be more efficient to have joint training and supervision protocols for these overlapping conditions. Future research could also explore the use of technology to scale up training and supervision, increasing the number of non-specialists trained in the delivery of such interventions, thus contributing to the reduction in the treatment gap for common mental disorder in the perinatal period.

## Conclusion

This new psychosocial intervention for perinatal anxiety, based on principles of CBT, was found to be acceptable and perceived to be helpful by the women who received it. The intervention was feasibly delivered by non-specialist professionals after brief classroom training and a few practice sessions under supervision. It therefore has the potential to address this important but neglected condition in LMICs. However, effectiveness studies are required prior to recommendations for its integration into perinatal care.

## Data Availability Statement

The datasets generated for this study are available on request to the corresponding author.

## Ethics Statement

The studies involving human participants were reviewed and approved by Institutional Review Board (IRB) of the Human Development Research Foundation. The patients/participants provided their written informed consent to participate in this study.

## Author Contributions

AR, NA, and PS designed the study. NA, PS, SZ, and HN carried out data analysis and interpretation. NA developed the intervention supervised by AR. The fieldwork was supported by RC and AM. MA translated the intervention materials into Urdu. NA wrote the first draft of the manuscript, and all authors assisted in critically reviewing it. NA and AR carried out the final revisions to the manuscript. All authors approved the final article.

## Funding

The research reported in this publication was supported by the National Institute of Mental Health of the National Institutes of Health under award number 3R01MH111859. The content is solely the responsibility of the authors and does not necessarily represent the official views of the National Institute of Mental Health, or the U.S. Department of Health and Human Services.

## Conflict of Interest

The authors declare that the research was conducted in the absence of any commercial or financial relationships that could be construed as a potential conflict of interest.
