# Supplementary material for: Development of a Psychological Intervention to Address Anxiety During Pregnancy in a Low-Income Country
Source: Front Psychiatry. 2020 Jan 10;10:927. doi: 10.3389/fpsyt.2019.00927 (PMC6967413; doi:10.3389/fpsyt.2019.00927)
Supplement: Supplementary file 1 [file DataSheet_1.pdf]

PSYCHLOPS: Pre-Therapy Version

Question 1

A: Choose the problem that troubles you most. *(Please write it in the box below).*

B: How much has it affected you over the last week? *(Please tick one box below).*

	0	1	2	3	4	5	
Not at all affected	☐	☐	☐	☐	☐	☐	Severely affected

C: How long ago were you first concerned about this problem? (*Please tick one box below).*

☐Under one month ☐ Between one and three months ☐ Over three months but under one year ☐ One to five years ☐ Over five year

Question 2

A: Choose another problem that troubles you. *(Please write it in the box below).*

B: How much has it affected you over the last week? *(Please tick one box below).*

	0	1	2	3	4	5	
Not at all affected	☐	☐	☐	☐	☐	☐	Severely affected

C: How long ago were you first concerned about this problem? (*Please tick one box below).*

☐Under one month ☐ Between one and three months ☐over three months but under one year ☐One to five years ☐ Over five years

Question 3

A: Choose one thing that is hard to do because of your problem (or problems). *(Please write it in the box below).*

B: How hard has it been to do this thing over the last week? *(Please tick one box below).*

	0	1	2	3	4	5	
Not at all hard	☐	☐	☐	☐	☐	☐	Very hard

Question 4

How have you felt about yourself this last week? *(Please tick one box below).*

	0	1	2	3	4	5	
Very good	☐	☐	☐	☐	☐	☐	Very bad

PSYCHLOPS: During-Therapy Version

Question 1

A: This is the problem you said troubled you the most when we first asked. *(Therapist - please write it in the box below).*

B: How much has it affected you over the last week? *(Please tick one box below).*

	0	1	2	3	4	5	
Not at all affected	☐	☐	☐	☐	☐	☐	Severely affected

Question 2

A: This is the other problem you said troubled you when we first asked. *(Therapist - please write it in the box below).*

B: How much has it affected you over the last week? *(Please tick one box below).*

	0	1	2	3	4	5	
Not at all affected	☐	☐	☐	☐	☐	☐	Severely affected

Question 3

A: This is the thing you said was hard to do when we first asked. *(Therapist - please write it in the box below).*

B: How hard has it been to do this thing over the last week? *(Please tick one box below).*

	0	1	2	3	4	5	
Not at all hard	☐	☐	☐	☐	☐	☐	Very hard

Question 4

How have you felt about yourself this last week? *(Please tick one box below).*

	0	1	2	3	4	5	
Very good	☐	☐	☐	☐	☐	☐	Very bad

Question 5

A: Now that we are participating in the program, you may have found that other problems have become important. If so, please write the one that troubles you most in the box below, or leave blank if no other problems have become important.

B: How much have these other problems affected you over the last week? *(Please tick one box below, or leave blank if no other problems have become important).*

	0	1	2	3	4	5	
Not at all affected	☐	☐	☐	☐	☐	☐	Severely affected

Comments

This box is provided for any comments you may want to record on the program so far.

PSYCHLOPS: Post-Therapy Version

Question 1

A. This is the problem you said troubled you the most when we first asked. *(Therapist -please write it in the box below)*

B. How much has it affected you over the last week? *(Please tick one box below).*

	0	1	2	3	4	5	
**Not at all affected**	☐	☐	☐	☐	☐	☐	**Severely affected**

Question 2

A. This is the other problem you said troubled you when we first asked (Therapist - please write it in box below).

B. How much has it affected you over the last week? (Please tick one box below).

	0	1	2	3	4	5	
**Not at all affected**	☐	☐	☐	☐	☐	☐	**Severely affected**

Question 3

A. This is the thing you said was hard to do when we first asked. (Therapist - please write it in the box below.)

B. How hard has it been to do this thing over the last week? (Please tick one box below).

	0	1	2	3	4	5	
**Not at all hard**	☐	☐	☐	☐	☐	☐	**Very hard**

Question 4

A. How have you felt in yourself this last week? (Please tick one box below).

	0	1	2	3	4	5	
**Very good**	☐	☐	☐	☐	☐	☐	**Very bad**

Question 5

During therapy, you may have found that other problems became important. If so, how much have these problems affected you over the last week?
(Please tick one box below, or leave blank if no other problems have become important).

	0	1	2	3	4	5	
**Not at all affected**	☐	☐	☐	☐	☐	☐	**Severely affected**

Question 6

Compared to when you started therapy, how do you feel now? (Please tick one box below).

0	1	2	3	4	5
☐	☐	☐	☐	☐	☐
Much better	Quite a lot better	A little better	About the same	A little worse	Much worse

This questionnaire is called the Psychological Outcome Profiles questionnaire (PSYCHLOPS), Post-Therapy Version 5.

Therapist Assessment Form – post therapy
To be completed by the therapist and attached to the completed questionnaire.

Participant ID:

Therapist ID:

Participant's due date:

Validation question
Now that the therapy has finished, how would you describe the client overall?
(Please tick one box below.)

**0**	**1**	**2**	**3**	**4**	**5**
☐	☐	☐	☐	☐	☐
Much better	Quite a lot better	A little better	About the same	A little worse	Much worse

Comments

This box is provided for any comments you may want to record on the therapy.

Scoring PSYCHLOPS

- PSYCHLOPS has been designed as a mental health outcome measure. As such, the pre-therapy score is compared with subsequent scores (during therapy and post-therapy). The difference is the 'change score'.
- All of the responses in PSYCHLOPS are scored on a six point scale ranging from zero to five. The higher the value, the more severely the person is affected.
- Not every question in PSYCHLOPS is used for scoring. Only the questions relating to Problems (Questions 1b and 2b), Functioning (Question 3b) and Wellbeing (Question 4) are scored. Other questions provide useful information but do not contribute to the change score.
- The questions used for scoring are indicated with the symbol: ___ This symbol appears after the scoring boxes. The therapist may find it helpful to insert the score inside this symbol.
- PSYCHLOPS therefore consists of three domains (Problems, Functioning and Wellbeing) and four questions which are scored.
- The maximum PSYCHLOPS score is 20.
- The maximum score for each question is 5.
- If both Q1 (Problem 1) and Q2 (Problem 2) have been completed, the total score is: Q1b + Q2b + Q3b + Q4.
- If Q1 (Problem 1) has been completed and Q2 (Problem 2) has been omitted, the total score is: (Q1b x 2) + Q3b + Q4. In other words, the score of Q1b (Problem 1) is doubled. This ensures that the maximum PSYCHLOPS score remains 20.

Total PSYCHLOPS Post-Therapy score: ______________________________
